# Exploring Depression among the Elderly during the COVID-19 Pandemic: The Effects of the Big Five, Media Use, and Perceived Social Support

**DOI:** 10.3390/ijerph192013534

**Published:** 2022-10-19

**Authors:** Yang Li, Zhi Lin, Yibo Wu

**Affiliations:** 1 College of Communication and Art Design, University of Shanghai for Science and Technology, Shanghai 200093, China; 2 School of Public Health, Peking University, Beijing 100191, China

**Keywords:** depression, perceived social support, Big Five, media use, physical health, the elderly

## Abstract

The mental health of the elderly is vulnerable during public health emergencies, such as the COVID-19 pandemic, and the risks of depression is increased. The study aimed to explore the effects of physical health, the Big Five personality traits, media use, and different kinds of perceived social support as predictors of levels of depression among the elderly during the COVID-19 pandemic. A cross-sectional survey was conducted in more than 120 cities in China with a sample of 1147 older adults, and hierarchical regression was established to examine the predictors of the level of depression. The results showed that almost half of the elderly (48%) had a status of mild or much more severe depression during the COVID-19 pandemic. The level of depression was negatively associated with physical health, extraversion, conscientiousness, agreeableness, and family support, while it was positively associated with neuroticism and media use. The study emphasized the effects of physical health, the Big Five personality traits, media use, and perceived social support from family as the predictors of levels of depression, providing further measures to help the elderly better react to a public health emergency.

## 1. Introduction

During public health emergencies, such as the coronavirus disease 2019 (COVID-19) pandemic, the risk of psychological problems among the public, such as depression, increases [1]. The related lockdown policies, social distancing, and lack of social activities might cause severe psychological problems [2]. The elderly suffered greater risks of infection because of their physical limitations during the COVID-19 pandemic compared to other age groups [3]. This situation creates more vulnerable mental and physical health in the elderly [4], making the elderly prone to feel more negative, depressive emotions. As the COVID-19 pandemic has become normalized, in order to take further actions to reduce the risk of depression among the elderly against the background of public health emergencies, exploring the predictors of depression is necessary. 

### 1.1. Physical Health and Depression

It is accepted that mental health has a close connection with physical health [5]. Chen and Austin demonstrated the association between self-rated physical health and depression [6]. The elderly are likelier to have physical diseases, such as cardiovascular disease, diabetes, or respiratory illness, increasing the risks of severe COVID-19 infection [7]. This leads to a greater panic about public health emergencies among the elderly, bringing about correlative depression. In other words, great physical health might reduce fear and worry in public health emergencies, such as the COVID-19 pandemic, which might be propitious for keeping depression at bay. 

### 1.2. The Big Five and Depression

The general patterns of individual differences, such as personality, may have an impact on how individuals respond to a public health emergency [8], and the emotional stability of personality traits is connected with physical health and mental health [9]. The Big Five personality traits are widely used in personality-related research, and refer to five central and stable personality traits: openness, conscientiousness, extraversion, agreeableness, and neuroticism [10]. 

Each specific Big Five personality trait showed different correlations with health outcomes. Extraversion was proven to be negatively associated with psychological problems and somatic health symptoms [11]. Conscientiousness, which implies being responsible and orderly-directed, had a reliably positive relationship with self-reported physical health [12] (p. 105) [13]. Low agreeableness seemed to be correlated with poor physical health [14]. High neuroticism was consistently related to a high risk of actual disease, including both physical and mental problems [15]. Finally, it was suggested that openness might have protective effects on physical illness [16]. Although some consensus has been reached on the association between each of the Big Five personality traits and specific health outcomes, the relevant results were not the same in different studies. 

For depression, these five kinds of personality traits might have distinctive influences, and they might cause different effects on different genders [17]. Carreira-Míguez et al. found that a depression group showed higher neuroticism, higher openness, and lower extraversion [18]. Koorevaar’s research among the elderly also demonstrated that depression was significantly correlated with higher neuroticism, lower extraversion, and lower conscientiousness, and higher openness had a statistically significant association with earlier stages of depression [19]. Research during the COVID-19 pandemic showed that more severe depressive symptoms were correlated with lower agreeableness [20]. It showed that the Big Five personality traits could provide more specific categories for exploring the effect of personality on depression. 

### 1.3. Media Use and Depression

Media use is an important component of modern daily life, and it is inherently personal. When focusing on how individuals use each kind of media, personal characteristics are crucial observational factors. It has been proven that the Big Five personality traits were associated with certain media use behaviors. For example, total Internet usage had negative associations with agreeableness, conscientiousness, and extraversion [21], while the overuse of a smartphone was related to neuroticism, conscientiousness, and agreeableness [22]. 

Media use can act imperceptibly on individuals’ mental health. During the COVID-19 pandemic, the mass media have played an important role in conveying health-related information, accessing perceived social support, and so on [23]. However, lockdown policies and possible concerns about the epidemic situation might lead to more problematic media use [24]. At the same time, COVID-19-related media use could have effects on depression [25]. 

In contrast with other age groups, the limitations of the physical and cognitive abilities of the elderly lead to them using smartphones less and digital media differently [26,27]. These limitations might cause different media preferences and different patterns of media repertoires between the elderly and other groups. This indicates that exploring the effect of the usage of media repertoires rather than just a single media type could help us better understand the media use of the elderly to some extent [28]. As a result, it is necessary to explore the effects of overall media use on the elderly’s mental health, examining different kinds of mass media, such as newspapers, magazines, books, radio, television, computers, and smartphones. 

### 1.4. Perceived Social Support and Depression

Perceived social support refers to the cognition and appraisal of individuals’ reliable connections with others [29], which is an essential component of social support [30]. Demographics, including gender, age, marital status, and each of the Big Five personality traits, are usually correlated with perceived social support [31,32,33]. 

Perceived social support has always been regarded as an important positive factor in both physical health and mental health [34,35], helping strengthen the resistance to depression [36]. In daily life, perceived social support can be obtained both offline and online. Using online social media to access perceived social support is an effective way to ward off the negative influences of health emergencies on mental health [37]. 

Against the background of the COVID-19 pandemic, the impact of perceived social support as a protective factor of mental health has attracted much attention. Preventive measures, such as home quarantine and maintenance of social distance, might reduce perceived social support, especially for the elderly [38], which increases the risk of depression. In terms of different sources, perceived social support consists of family support, friends’ support, and support from significant others [39]. Perceived social support from friends and significant others decreases with age [31]. Therefore, for the elderly, different sources of perceived social support may have different degrees of effects on predicting depression. 

Based on the above discussion, this study examines the predictive power of physical health, Big Five personality, media use, and different sources of perceived social support on depression among the elderly during the COVID-19 pandemic. The research questions and hypotheses were formulated as follows.

**RQ1:**  *Was physical health significantly related to the level of depression among the elderly during the COVID-19 pandemic?*

**RQ2:**  *Were the Big Five personality traits significantly related to the level of depression among the elderly during the COVID-19 pandemic?*

**H1:**  *Extraversion is negatively associated with the level of depression.*

**H2:**  *Conscientiousness is negatively associated with the level of depression.*

**H3:**  *Agreeableness is negatively associated with the level of depression.*

**H4:**  *Neuroticism is positively associated with the level of depression.*

**H5:**  *Openness is negatively associated with predicting the level of depression.*

**RQ3:**  *Was overall media use significantly related to the level of depression among the elderly during the COVID-19 pandemic?*

**RQ4:**  *Was perceived social support significantly related to the level of depression among the elderly during the COVID-19 pandemic?*

**H6:**  *Family support is negatively associated with the level of depression.*

**H7:**  *Friends’ support is negatively associated with the level of depression.*

**H8:**  *Significant others’ support is negatively associated with the level of depression.*

The demographic factors that might be correlated with the level of depression [40,41,42], such as gender, age, income, and marital status, were also included for examination in the study. 

## 2. Materials and Methods

### 2.1. Sampling

A cross-sectional survey using multistage sampling was conducted in China from 10 July to 15 September in 2021, which was during the COVID-19 pandemic. According to the Seventh National Population Census of the People’s Republic of China in 2021, quota sampling (quota attributes: gender, age, and urban–rural distribution) of the selected elderly residents from 120 cities was finished. 

The participants had to meet the following criteria: (1) aged 60 years old or older; (2) Chinese nationality (from the People’s Republic of China); (3) willingness to participate in the study voluntarily; (4) ability to complete the network questionnaire survey on their own or with the help of the investigators; and (5) ability to understand the meaning of each question in the questionnaire. A total of 1147 completed questionnaires were valid for the study. 

### 2.2. Measurement

The questionnaire consisted of two parts. The first part was the demographic characteristics (gender, age, per capita monthly household income, marital status), and the second part included scales that were utilized to measure depression, physical health, personality, media use, and perceived social support. 

#### 2.2.1. Depression Status

To measure the level of depression symptoms among the elderly, the Patient Health Questionnaire (PHQ-9) was employed, as it is effective for the evaluation of depression disorder [43]. The PHQ-9 is a 4-point rating scale, with the response options ranging from “not at all (=0)” to “nearly every day (=3)”. The scores on the PHQ-9 ranged between 0 and 27: 0–4 indicated minimal depression, 5–9 indicated mild depression, 10–14 indicated moderate depression, 15–19 indicated moderately severe depression, and 20–27 indicated severe depression [43]. The Cronbach’s α of PHQ-9 in the current study was 0.930.

#### 2.2.2. Physical Health

The EQ-5D-5L is a generic instrument to assess individual health, which includes dimensions of mobility, self-care, usual activities, pain or discomfort, and anxiety [44]. In the current study, the first four items of the EQ-5D-5L were used to measure the level of physical health because the study utilized the PHQ-9 to measure the dependent variable depression, which was similar to the fifth dimension to some extent. EQ-5D-5L usually used a valuation set to indicate the level of health, but there was no valuation set for EQ-4D-5L. As a result, these four items measuring the level of physical health in the study were rated on a scale, take the first item for example, (1) I am unable to walk about (=1); (2) I have slight problems in walking about (=2); (3) I have moderate problems in walking about (=3); (4) I have severe problems in walking about (=4); (5) I have no problems in (=5). The scores of EQ-4D-5L ranged from 4 to 20, and the higher scores indicated a better condition of physical health. The Cronbach’s α of the EQ-4D-5L in the study was 0.871.

#### 2.2.3. Personality

A 10-item short version of the Big Five Inventory (BFI-10) was employed to measure the main personality traits of the elderly, including five dimensions: openness, conscientiousness, extraversion, agreeableness, and neuroticism. This was a reliably abbreviated version of the BFI-44. The BFI-10 used a 5-point Likert scale ranging from 1 (totally disagree) to 5 (totally agree) [45]. The score for each dimension ranged between 2 and 10, and higher scores indicated stronger tendencies. 

#### 2.2.4. Media Use

Media use was measured using the following question: How often do you use [newspapers, magazines, books, radio, television, computers, smartphones] in daily life each week? (1) never use (=1); (2) ≤1 day/week (=2); (3) 2–3 days/week (=3); (4) 4–5 days/week (=4); (5) 6–7 days/week (=5). Cronbach’s α of media use was 0.753 in this study. The scores for each kind of media were added to measure overall media use. The total score ranged from 7 to 35, and higher scores indicated more media use. The descriptive statistics of the use frequency of different kinds of media are presented in Appendix A (Table A1). 

#### 2.2.5. Perceived Social Support

The Multidimensional Scale of Perceived Social Support (MSPSS) was applied to “assess perceptions of social support adequacy from three specific sources [to] measure the basic figures of individual personality” [39]. The MSPSS included 12 items, which could be divided into three dimensions: family support, friends’ support, and significant other’s support [39]. The score for each dimension of the MSPSS ranged from 4 to 28, and higher scores indicated higher perceived social support. The internal reliability measured by Cronbach’s α was 0.88 in the original literature and 0.952 in the present study. The present study examined the effects of three dimensions of the MSPSS on depression among the elderly. The Cronbach’s α of each dimension (family support, friends’ support, significant other’s support) were 0.905, 0.913, and 0.897, respectively. 

### 2.3. Statistical Analysis

The study utilized SPSS 25.0 to conduct the statistical analysis. First, descriptive statistics were conducted to examine the demographic characteristics of the participants in the current study. Correlation analysis and hierarchical regression analyses were then utilized to analyze the relationship between depression and the Big Five personality traits, media exposure, and different sources of perceived social support.

## 3. Results

### 3.1. Descriptive Statistics

In total, 1147 participants were recruited for the present study (Table 1), including 581 males (50.65%) and 566 females (49.35%). The results of the descriptive statistics showed that most of the participants were 60–80 years old. More than 60% of participants (61.82%) reported that their per capita monthly household income was less than 4500 yuan, and only 8.10% reported that their per capita monthly household income was more than 9000 yuan. Most of the participants were married (77.94%), and 16.74% were widowed. According to the scores of PHQ-9, 52% of participants had minimal depression, 31.56% had mild depression, 10.46% had moderate depression, 5.14% had moderately severe depression and 0.87% had severe depression. 

The median scores and inter-quartile range (IQR) of specific scales were as follows: PHQ-9, 4.00 (1.00–8.00); EQ-4D-5L (used to measure the physical health of the elderly), 19.00 (18.00–20.00); openness, 6.00 (5.00–7.00); conscientiousness, 7.00 (6.00–8.00); extraversion, 6.00 (5.00–7.00); agreeableness, 7.00 (6.00–8.00); neuroticism, 6.00 (5.00–6.00); media use, 17.00 (13.00–21.00); significant other’s support, 20.00 (17.00–24.00); family support, 21.00 (17.00–24.00); and friends’ support, 20.00 (16.00–24.00). 

### 3.2. Correlation Analysis

The Spearman correlation coefficient was utilized to examine the correlation between related factors, and the results are shown in Table 2. Depression had a significantly negative association with physical health (r = −0.422, *p* < 0.001), conscientiousness (r = −0.266, *p* < 0.001), extraversion (r = −0.118, *p* < 0.001), agreeableness (r = −0.271, *p* < 0.001), significant other’s support (r = −0.267, *p* < 0.001), family support (r = −0.319, *p* < 0.001) and friend’s support (r = −0.247, *p* < 0.001). In addition, depression had a significantly positive association with neuroticism (r = 0.286, *p* < 0.001) and media use (r = 0.084, *p* < 0.01). There was no significant association between openness and depression (r = 0.001, *p* > 0.05). 

### 3.3. Hierarchical Regression

Hierarchical regression was conducted to examine the predictors of depression among the elderly during the public health emergency (Table 3). The regression model included gender, age, per capita monthly household income, marital status, and physical health in the first model (step 1) (R^2^_Adjusted_ = 0.148, *p* < 0.001). The Big Five personality traits (openness, conscientiousness, extraversion, agreeableness, neuroticism) were added in the second model (step 2) (R^2^_Adjusted_ = 0.248, *p* < 0.001). Media use was then added in step 3 (R^2^_Adjusted_ = 0.289, *p* < 0.001), and three dimensions of perceived social support (family support, friend’s support, significant other’s support) were added in the final model (step 4) (R^2^_Adjusted_ = 0.304, *p* < 0.001). The residual error was approximately normally distributed. 

In the final model (step 4), gender (B = 0.448, *p* > 0.05), age (B = 0.018, *p* > 0.05), per capita monthly household income (B = −0.096, *p* > 0.05) and marital status (B = 0.259, *p* > 0.05) were not predictors of depression. Physical health could negatively predict the level of depression (B = −0.727, *p* < 0.001). For the Big Five personality traits, conscientiousness (B = −0.372, *p* < 0.001), extraversion (B = −0.202, *p* < 0.05) and agreeableness (B = −0.348, *p* < 0.001) were the negative predictors of depression, neuroticism (B = 0.389, *p* < 0.001) was a positive predictor of depression, and openness (B = 0.088, *p* > 0.05) could not predict the level of depression. Media use (B = 0.216, *p* < 0.001) could positively predict the level of depression. For different sources of perceived social support, family support (B = −0.205, *p* < 0.001) was a negative predictor of depression, while friends’ support (B < 0.001, *p* > 0.05) and significant other’s support (B = 0.041, *p* > 0.05) were not statistically significant indicators of depression. 

As a result, RQ1, RQ2, RQ3, and RQ4 were answered. Physical health showed protective effects against depression. The Big Five personality traits were significantly associated with depression, and H1, H2, H3, and H4 were supported while H5 was rejected. Media use showed a positive association with depression. In terms of different sources of perceived social support, only family support showed a significantly negative association with depression; H6 was supported, and H7 and H8 were rejected. 

## 4. Discussion

The present study examined the effects of the Big Five personality traits, media use, and perceived social support on depression in the elderly using hierarchical regression. The results revealed that physical health, conscientiousness, extraversion, agreeableness, and family support were significantly negatively associated with depression. Neuroticism and media use had significantly positive associations with depression. The study used a sample of 1147 Chinese elderly people gathered through multistage sampling covering 120 cities in China, which yielded representative results that reduced random errors to some extent. According to the descriptive data, almost half of the elderly (48%) had a status of mild or much more severe depression during the COVID-19 pandemic period. 

Physical health still showed a protective role in the development of depression. It indicated the importance of maintaining physical health. It is known that physical health has a negative correlation with age, and the elderly have lower levels of physical health [46]. Engaging in physical activity is an effective method of promoting physical health and reducing depressive symptoms [47], and for the elderly, proper physical activity is crucial. The *WHO guidelines on physical activity and sedentary behavior* provide authoritative reference content in this matter [48]. 

In terms of the Big Five personality traits, conscientiousness, extraversion, agreeableness, and neuroticism were predictive factors of depression in the elderly. On the one hand, conscientiousness, extraversion, and agreeableness showed significant associations with the level of depression. This was different from the results of Koorevaar’s research, in which agreeableness did not show a significant association with depression [19]. This might be because of the different measurement tools used for the Big Five and depression, and different kinds of participants. Koorevaar used the NEO-Five Factor Inventory (NEO-FFI) and the Inventory of Depressive Symptomatology (IDS), while 10 items of the Big Five Inventory (BFI-10) and the Patient Health Questionnaire (PHQ-9) were employed in the study. In addition, the patient group was required to have a diagnosis of depression in Koorevaar’s research, while the current research did not set this inclusion criterion. On the other hand, neuroticism had a significantly positive impact on depression, which was the same as in previous research [18,19,20]. These results in the current study remind us to take better care of the elderly with higher neuroticism and lower extraversion, agreeableness, and conscientiousness. Elderly people with higher neuroticism are likelier to experience negative emotions, such as worry and anxiety, especially when facing a public health emergency. Low extraversion could indicate a low activity level, low agreeableness implies not being close to people, and low conscientiousness means a low self-discipline level [49] (pp. 206–210). These features are averse to maintaining physical health or obtaining perceived social support, increasing the risk of depression among the elderly. Although the personality traits of the elderly are stable and hard to change [50], understanding the personality traits of the elderly to take preventive measures in advance is still a feasible measure for protecting them from possible depression. 

Media use also had an effect on depression among the elderly during the COVID-19 pandemic period. Previous research focused on COVID-19-related media exposure indicated a negative association between media use and mental health [51]. The current study investigated the effect of media use and showed that more frequent media use would increase the rate of depression. The importance of moderate use of various media types was emphasized. Although people can obtain health-related information, perceived social support and use mHealth intervention by using media [52], there is a great deal of confusing content on the media during a public health emergency [53]. Especially for the elderly, the limitation of cognitive ability may make it difficult to distinguish useful media content. It brings about the negative effects of media use on mental health. Controlling the time of media use and guiding the proper use of media content and functions, such as keeping in touch with family or friends or identifying scientific health-related information, are important.

It was interesting that not all sources of perceived social support could protect the elderly from depression. The results indicated that only family support demonstrated a significant inverse association with depression. Compared to youth and younger adults, the elderly perceived lower support [54]. Perceived social support from their families was more useful for protecting them from depression. Family members should pay more attention to the elderly in the family and keep in touch with them, giving them careful support. Combined with the above discussions, family members could help the elderly conduct physical examinations on time, take necessary preventive actions according to their personalities, and guide them to use media properly to reduce the risk of depression when a public health emergency occurs. 

This study emphasized the significance of paying attention to depression in the elderly under the background of health emergencies. To take further measures to help the elderly better react to possible depression during a public health emergency, especially a normalized epidemic, focusing on the characteristics and habits of the elderly could provide some coping strategies. 

## 5. Conclusions

The present study conducted a hierarchical regression model to examine the predictive power of physical health, the Big Five personality traits, media use, and three different kinds of perceived social support for the level of depression among the elderly during a public emergency. The results showed that depression was negatively associated with physical health, extraversion, conscientiousness, agreeableness, and family support, while it was positively associated with neuroticism and media use. 

The study also provided some coping insights for protecting the elderly from possible depression during a public health emergency, especially in the current normalized epidemic period. To protect the elderly from depression or lower the level of their depression during public health emergencies, adopting different guidance based on different personality traits of the elderly can provide advanced monitoring. Second, engaging in proper physical activity or physical examinations on time to maintain physical health can strengthen the physical basis for preventing psychological problems. In addition, focusing on media use and guiding the elderly to use media properly or learn beneficial functions are all necessary. Moreover, families should take better care of the elderly in the family, as perceived social support from the family is a forceful power to protect them from depression during public health emergencies. 

In the study, the 10-item short version of the Big Five Inventory (BFI-10) was utilized to measure personality traits among the elderly, but further studies could utilize the scale containing more items to measure the Big Five personality traits, such as the 44 items of the Big Five Inventory (BFI-44), or try to examine the association between more multidimensional personality traits and depression. In addition, the correlation between further media use, such as the preferences for media content and depression is valuable to explore. 

## Figures and Tables

**Table 1:** Descriptive statistics of participants.

Variable	N (%)/Median (IQR)
Gender	
Male	581 (50.65)
Female	566 (49.35)
Age group	
60–65	272 (23.71)
66–70	242 (21.10)
71–75	261 (31.47)
76–80	164 (14.30)
81–85	73 (6.36)
86–90	23 (2.00)
≥91	12 (1.05)
Per capita monthly household income (yuan)	
≤1500	211 (18.40)
1501–3000	260 (22.67)
3001–4500	238 (20.75)
4501–6000	168 (14.65)
6001–9000	177 (15.43)
9001–12,000	48 (4.18)
≥12,001	45 (3.92)
Marital status	
Married	894 (77.94)
Unmarried	41 (3.57)
Divorced	20 (1.74)
Widowed	192 (16.74)
Physical health (EQ-4D-5L)	19.00 (18.00–20.00)
Openness	6.00 (5.00–7.00)
Conscientiousness	7.00 (6.00–8.00)
Extraversion	6.00 (5.00–7.00)
Agreeableness (mean ± SD)	7.00 (6.00–8.00)
Neuroticism (mean ± SD)	6.00 (5.00–6.00)
Media use (mean ± SD)	17.00 (13.00–21.00)
Significant other support (mean ± SD)	20.00 (17.00–24.00)
Family support (mean ± SD)	21.00 (17.00–24.00)
Friends support (mean ± SD)	20.00 (16.00–24.00)
Depression (PHQ-9) (mean ± SD)	4.00 (1.00–8.00)
Minimal depression	596 (51.96)
Mild depression	362 (31.56)
Moderate depression	120 (10.46)
Moderately severe depression	59 (5.14)
Severe depression	10 (0.87)

**Table 2:** Correlation analysis between the scores of PHQ-9 and the other variables.

Variable	1	2	3	4	5	6	7	8	9	10	11
Depression	1	0.001	−0.266 ***	−0.118 ***	−0.271 ***	0.286 ***	0.084 **	−0.267 ***	−0.319 ***	−0.247 ***	−0.422 ***
Openness		1	0.096 *	0.228 ***	0.058 *	−0.061 *	0.218 ***	0.048	0.069 *	0.114 ***	0.065 *
Conscientiousness			1	0.210 ***	0.432 ***	−0.270 ***	−0.052	0.412 ***	0.420 ***	0.324 ***	0.067 *
Extraversion				1	0.037	−0.134 ***	0.059 *	0.150 ***	0.146 ***	0.164 ***	0.093 **
Agreeableness					1	−0.359 ***	0.044	0.388 ***	0.408 ***	0.369 ***	0.104 ***
Neuroticism						1	−0.054	−0.238 ***	−0.272 ***	−0.229 ***	−0.138 ***
Media use							1	0.045	0.085 **	0.133 ***	0.099 **
Significant other support								1	0.834 ***	0.845 ***	0.098 *
Family support									1	0.783 ***	0.159 ***
Friends support										1	0.139 ***
Physical health											1

* *p* < 0.05; ** *p* < 0.01; *** *p* < 0.001.

**Table 3:** The results of hierarchical regression.

	B	SE	β	R^2^_Adjusted_
Step 1				0.148 ***
Gender	0.054	0.282	0.005	
Age	−0.067	0.109	−0.018	
Marital status	−0.128	0.183	−0.020	
per capita monthly household income	0.086	0.086	0.028	
Physical health	−0.852 ***	0.061	0.398 ***	
Step 2				0.248 ***
Gender	0.120	0.268	0.012	
Age	−0.052	0.102	−0.014	
Marital status	0.052	0.174	0.008	
per capita monthly household income	0.074	0.082	0.024	
Physical health	−0.742 ***	0.058	0.346 ***	
Openness	0.239 *	0.095	0.068 *	
Conscientiousness	−0.552 ***	0.099	−0.165 ***	
Extraversion	−0.204 *	0.092	−0.061 *	
Agreeableness	−0.432 ***	0.102	−0.126 ***	
Neuroticism	0.433 ***	0.102	0.121 ***	
Step 3				0.289 ***
Gender	0.392	0.262	0.038	
Age	−0.001	0.100	<0.001	
Marital status	0.273	0.171	0.042	
per capita monthly household income	−0.094	0.082	−0.030	
Physical health	−0.746 ***	0.057	0.348 ***	
Openness	0.095	0.094	0.027	
Conscientiousness	−0.504 ***	0.096	−0.151 ***	
Extraversion	−0.226 *	0.089	−0.067 *	
Agreeableness	−0.463 ***	0.099	0.124 ***	
Neuroticism	0.443 ***	0.099	0.124 ***	
Media use	0.199 ***	0.024	0.226 ***	
Step 4				0.304 ***
Gender	0.448	0.260	0.044	
Age	0.018	0.099	0.005	
Marital status	0.259	0.171	0.040	
per capita monthly household income	−0.096	0.081	−0.060	
Physical health	−0.727 ***	0.056	0.339 ***	
Openness	0.088	0.093	0.025	
Conscientiousness	−0.372 ***	0.099	−0.111 ***	
Extraversion	−0.202 *	0.089	−0.060 *	
Agreeableness	−0.348 ***	0.101	−0.102 ***	
Neuroticism	0.389 ***	0.098	0.108 ***	
Media use	0.216 ***	0.024	0.245 ***	
Significant other support	0.041	0.066	0.035	
Family support	−0.205 ***	0.054	−0.177 ***	
Friends support	<0.001	0.058	<0.001	

* *p* < 0.05; *** *p* < 0.001.

## Data Availability

Data is contained within the article.

## Appendix A

**Table A1:** Descriptive statistics of use frequency of different kinds of media (media use).

Variable	Total (%)	Variable	Total (%)
Newspapers		Television	
never use	532 (46.38)	never use	64 (5.58)
1 day/week	212 (18.48)	1 day/week	112 (9.76)
2–3 days/week	190 (16.56)	2–3 days/week	191 (16.65)
4–5 days/week	142 (12.38)	4–5 days/week	281 (24.50)
6–7 days/week	71 (6.19)	6–7 days/week	499 (43.50)
Magazines		Computers (including panel computers)	
never use	657 (57.28)	never use	703 (61.29)
1 day/week	211 (18.40)	1 day/week	146 (12.73)
2–3 days/week	156 (13.60)	2–3 days/week	142 (12.38)
4–5 days/week	89 (7.76)	4–5 days/week	97 (8.46)
6–7 days/week	34 (2.96)	6–7 days/week	59 (5.14)
Books		Smartphones	
never use	529 (46.12)	never use	322 (28.07)
1 day/week	218 (19.01)	1 day/week	110 (9.59)
2–3 days/week	211 (18.40)	2–3 days/week	186 (16.22)
4–5 days/week	130 (11.33)	4–5 days/week	180 (15.69)
6–7 days/week	59 (5.14)	6–7 days/week	349 (30.43)
Radio			
never use	466 (40.63)		
1 day/week	202 (17.61)		
2–3 days/week	200 (17.44)		
4–5 days/week	186 (16.22)		
6–7 days/week	93 (8.11)		
